# Natural and Induced Humoral Responses to MUC1

**DOI:** 10.3390/cancers3033073

**Published:** 2011-07-29

**Authors:** Silvia von Mensdorff-Pouilly, Maria Moreno, René H. M. Verheijen

**Affiliations:** 1 Department of Obstetrics and Gynecology, VU University Medical Center, De Boelelaan 1117, Amsterdam 1081 HV, The Netherlands; E-Mail: mmoreno@higiene.edu.uy; 2 Department of Woman & Baby, Division of Surgical & Oncological Gynaecology, University Medical Center Utrecht, Heidelberglaan 100, Utrecht 3508 GA, The Netherlands; E-Mail: R.Verheijen@umcutrecht.nl

**Keywords:** natural humoral response, anti-MUC1 antibodies, MUC1-based vaccines, immunotherapy, cancer

## Abstract

MUC1 is a membrane-tethered mucin expressed on the ductal cell surface of glandular epithelial cells. Loss of polarization, overexpression and aberrant glycosylation of MUC1 in mucosal inflammation and in adenocarcinomas induces humoral immune responses to the mucin. MUC1 IgG responses have been associated with a benefit in survival in patients with breast, lung, pancreatic, ovarian and gastric carcinomas. Antibodies bound to the mucin may curb tumor progression by restoring cell-cell interactions altered by tumor-associated MUC1, thus preventing metastatic dissemination, as well as counteracting the immune suppression exerted by the molecule. Furthermore, anti-MUC1 antibodies are capable of effecting tumor cell killing by antibody-dependent cell-mediated cytotoxicity. Although cytotoxic T cells are indispensable to achieve anti-tumor responses in advanced disease, abs to tumor-associated antigens are ideally suited to address minimal residual disease and may be sufficient to exert adequate immune surveillance in an adjuvant setting, destroying tumor cells as they arise or maintaining occult disease in an equilibrium state. Initial evaluation of MUC1 peptide/glycopeptide mono and polyvalent vaccines has shown them to be immunogenic and safe; anti-tumor responses are scarce. Progress in carbohydrate synthesis has yielded a number of sophisticated substrates that include MUC1 glycopeptide epitopes that are at present in preclinical testing. Adjuvant vaccination with MUC1 glycopeptide polyvalent vaccines that induce strong humoral responses may prevent recurrence of disease in patients with early stage carcinomas. Furthermore, prophylactic immunotherapy targeting MUC1 may be a strategy to strengthen immune surveillance and prevent disease in subjects at hereditary high risk of breast, ovarian and colon cancer.

## 1. MUC1 Mucin

MUC1 mucin, encoded by the *MUC1* gene, is a membrane-bound glycoprotein expressed at the apical cell surface of normal secretory epithelia, as well as at the cell surface of some hematopoietic cells [1-3]. The molecule was first defined by murine monoclonal antibodies (MAb) generated against human milk fat globules, directed to a strongly immunogenic domain, the PDTR sequence, which is expressed multiple times on the extracellular MUC1 domain [4]. Since then, a number of MAbs to the same and other peptide and glycopeptide epitopes expressed on the molecule have been generated [5]. MUC1 is overexpressed and aberrantly glycosylated in carcinomas and in certain hematological malignancies [6-12]. High levels of MUC1 expression, particularly in the cytoplasm, are associated with a poor prognosis in a variety of carcinomas [13-17]. MUC1 is shed into the circulation, and is elevated in the serum of carcinoma patients. MUC1 serum levels (CA 15.3 and CA 15.3-like assays) are used to monitor response to treatment in patients with breast cancer, and in the follow up to detect disease recurrence [18,19].

**Figure 1.**
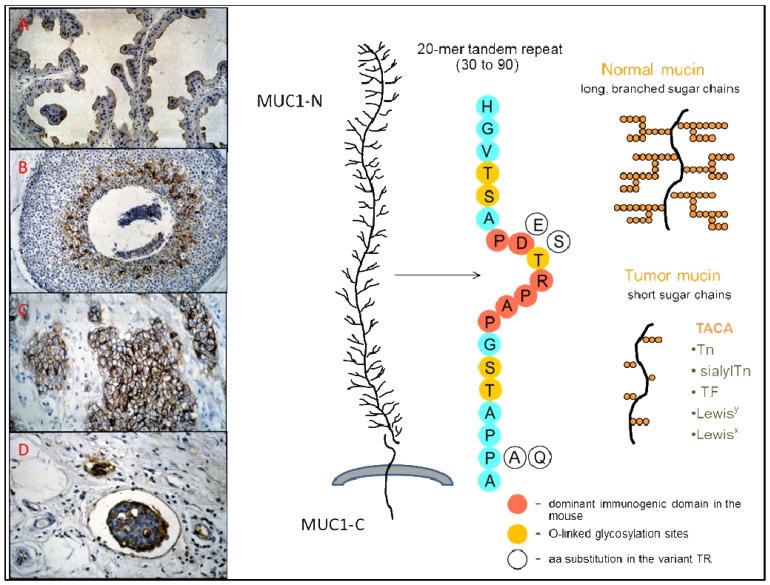
To the right of the figure, a schematic representation of the MUC1 molecule, the variant and nonvariant tandem repeat that form the major part of MUC1-N, and its glycosylation in normal and tumor-associated mucin. To the left of the figure, immunohistochemistry showing the expression of underglycosylated MUC1 in (**A**) apocrine metaplasia of the breast (MAb VU-4-H5); (**B**) ductal carcinoma *in situ* of the breast (MAb SM3); (**C**) invasive ductal adenocarcinoma of the breast (MAb VU-4-H5); (**D**) capillary with tumor embolus from an invasive ductal adenocarcinoma of the breast (MAb B27.29).

MUC1 is a high-molecular-weight (>400 kD) type I membrane-tethered glycoprotein with a C-terminal transmembrane subunit (MUC1-C) non-covalently bound to a large, highly glycosylated, N-terminal extracellular domain (MUC1-N) that consists mainly of numerous peptide repeats (TR), varying in number among the different alleles (Figure 1) [20-22]. These TR are present as tandem sequences of 20 amino-acids with five potential sites for O-linked glycosylation that are variably occupied and have shorter glycans in the tumor-associated mucin [1,23]. Variations in the highly conserved TR sequence occur at the level of the immunodominat PDTR domain: blocks of PDTR repeats are interspersed with blocks of PESR repeats, the latter being more numerous in the longer alleles [24,25]. Aberrant glycosylation of the extracellular domain of tumor-associated MUC1 leads to the exposure of immunogenic core peptide epitopes, and to the presence of tumor-associated carbohydrate antigens (TACA), such as the blood-group-related antigens Tn, sialylTn, and the Thomsen-Friedenreich (TF or T) antigen [26,27]. Together with the exposed core peptides, the restrictive distribution in normal tissues of TACA expressed on MUC1 and their extensive expression in epithelial cancer make them good targets for immunotherapy [28-31].

Further than the most obvious functions common to all mucins of lubricating epithelial cell surfaces, protecting them against irritants, and providing a barrier against infection, MUC1 is involved in epithelial sheet formation and morphogenesis, embryo implantation, cell-cell interaction, intracellular signaling and immune regulation [2,32-34]. In tumor cells, MUC1 functions as an oncoprotein and is involved directly or indirectly in most of the hallmarks of cancer either through the extracellular (MUC1-N) or the transmembrane (MUC1-C) subunit [2,35]. MUC1-N has immunosuppressive properties, and its overexpression on tumor cells strongly alters their adhesive properties favoring tumor progression and metastases [33,36,37]. The MUC1-C subunit is involved in several cellular signaling pathways that induce transformation and promote growth and survival of tumors, and activates genes involved in invasion, angiogenesis and metastases [38-40]. A signature of 35 MUC1-induced genes associated with oncogenesis, angiogenesis and extracellular matrix remodeling predict a poor disease outcome in breast and lung cancer patients [41]. The molecular characteristics of tumor-associated MUC1, its overexpression in virtually all adenocarcinomas and its functions as an oncoprotein make it a good target for the immunotherapy of cancer.

## 2. Natural Antibodies to MUC1

Natural antibodies (abs) directed to MUC1-N are present in the circulation of healthy individuals and patients with benign and malignant disease, either free or bound into immune complexes with MUC1 [42]. Several physiological and benign conditions are associated with altered expression of MUC1 that lead to immune responses to the antigen. MUC1 expression is increased in the breast during pregnancy and lactation. MUC1 serum levels are increased during pregnancy and, similar to ovarian cancer, the circulating mucin is less heavily sialylated than the mucin derived from normal tissues [43,44]. Pregnancy and lactation induce an immunologic response against MUC1 that could play a role in the mechanisms involved in the association between multiparity and a lower risk of breast cancer [45,46]. MUC1 is upregulated in response to chronic inflammation of the gastrointestinal tract, and expression of underglycosylated MUC1 is present in benign and premalignant lesions of several organs [47,48]. In carcinomas, cryptic core peptide epitopes on the MUC1 molecule shed into the tumor microenvironment and the circulation induce humoral and cellular immune responses to the mucin [49,50]. B-cells from tumor draining lymph nodes from ovarian cancer patients produce abs that react with the MUC1 protein core [49]. A significant correlation was found between the ability to isolate MUC1-specific B cells from tumor-draining lymph nodes and the presence of circulating abs to MUC1 [51].

### 2.1. MUC1 Immune Complexes

Circulating MUC1 immune complexes containing IgM and/or IgG have been described in the serum from pregnant and lactating women, in patients with benign breast tumors, and in patients with carcinoma of the breast, ovary, and head and neck [46,52-55]. A negative correlation between MUC1 and MUC1 immune complex serum levels has been described in the majority of these studies. In 140 breast cancer patients, the presence of MUC1 immune complexes in serum samples obtained before primary treatment was inversely correlated to stage of disease at diagnosis. None of the patients with pretreatment elevation of both MUC1 immune complexes and MUC1 (N = 13) relapsed during the observation period. Furthermore, 23/28 patients (82%) that relapsed during the observation period were MUC1 immune complex negative, suggesting that a humoral immune response to MUC1 may protect against disease progression [56].

### 2.2. Free Antibodies to MUC1

Free circulating abs to MUC1 were first described in ulcerative colitis, a chronic inflammatory disorder of the colon. The reactivity of 19 patients' sera with a recombinant MUC1 protein containing 10 tandem repeats was evaluated by SDS-PAGE and Western blot analysis. The reactivity of the five positive serum samples with the recombinant protein was completely inhibited by a monoclonal antibody (MAb) to the tandem repeat [57]. High levels of IgG to purified colonic mucins were detected in patients with chronic continuous and relapsing-remitting type ulcerative colitis but not in patients who had had only one attack with no relapses [58]. Circulating anti-colon antibodies to mucins and other cellular components of the colonic epithelium may arise as a consequence of or lead to colonic mucosa inflammation, and could be involved in the pathogenesis of ulcerative colitis [59]. Gastric mucins are altered in individuals infected with *Helicobacter pylori (H. pylori)* [47]. MUC1 IgG and IgM ab levels were studied in 91 healthy blood donors, 160 patients with chronic gastroduodenal diseases and in 214 patients with gastric cancer and related to *H. pylori* serologic status. Higher levels of MUC1 IgG abs were found in *H. pylori* seropositive patients with benign disease (*p* < 0.01) and healthy donors (*p* < 0.03) than in *H. pylori* seronegative subjects, and were associated with a higher degree of gastric corpus mucosa inflammation in patients with chronic gastroduodenal diseases (*p* < 0.0025). MUC1 IgM abs were not related to *H. pylori* serology. Patients with gastric cancer had higher levels of MUC1 IgG abs than blood donors (*p* < 0.001) irrespective of *H. pylori* serologic status or stage of disease. The findings suggest that *H. pylori* infection may induce an immune response to tumor-associated MUC1 [60].

Circulating MUC1 IgM abs reactive with the MUC1-N protein core are present in breast, colon and pancreatic cancer patients [61], as well as in healthy women and benign and malignant ovarian tumor patients [62]. Samples were tested with an enzyme-linked immunosorbent assay (ELISA) and abs captured with a 105-mer synthetic MUC1 peptide corresponding to >5 MUC1-N tandem repeats. Concentrations of MUC1 IgM abs in healthy women were inversely correlated to age, and were higher than in ovarian cancer patients, regardless of age. MUC1 IgM ab levels were higher in patients with serous cystadenomas than in patients with mucinous cystadenomas. A negative correlation between MUC1 IgM abs and MUC1 antigen levels was observed, suggesting the formation of immune complexes with MUC1. Although high ab levels were associated with a greater survival in ovarian cancer, they had no independent prognostic value [62]. No IgG responses to MUC1 were detected in these two studies.

We developed a robust ELISA based on a synthetic triple MUC1 TR coupled to BSA that was standardized initially against a serum sample from two subjects with high MUC1 IgG or IgM abs, and later against a humanized MUC1 IgG1 MAb (huHMFG1) and a human IgM MAb (2F8) for IgG and IgM responses, respectively. The sensitivity of the assay is high: screening dilutions were set at 1:100 for IgG and at 1:500 for IgM determinations [63,64]. In contrast to the findings of Richards and colleagues [62] we found no correlation between MUC1 IgM (or IgG) abs and age in the total population (N 456) or in the groups studied (40 healthy men, 101 healthy women, 54 nulligravidae, 45 pregnant women, 62 patients with benign breast tumors, and 154 patients with early breast cancer). In agreement with Croce and colleagues [46], pregnant women had significantly lower levels of MUC1 IgM abs (*p* < 0.0001) than all other groups studied. On the other hand, nulligravidae had the lowest levels of MUC1 IgG abs as compared with all other groups studied (*p* < 0.01). Benign breast tumor and early breast cancer patients had significantly higher levels of MUC1 IgG abs than healthy women (*p* = 0.0036 and *p* = 0.0124, respectively), but the levels did not differ significantly between them. In contrast to other studies, we found no correlation between MUC1 IgG or IgM ab levels and MUC1 (CA 15.3) assay results [65].

**Figure 2.**
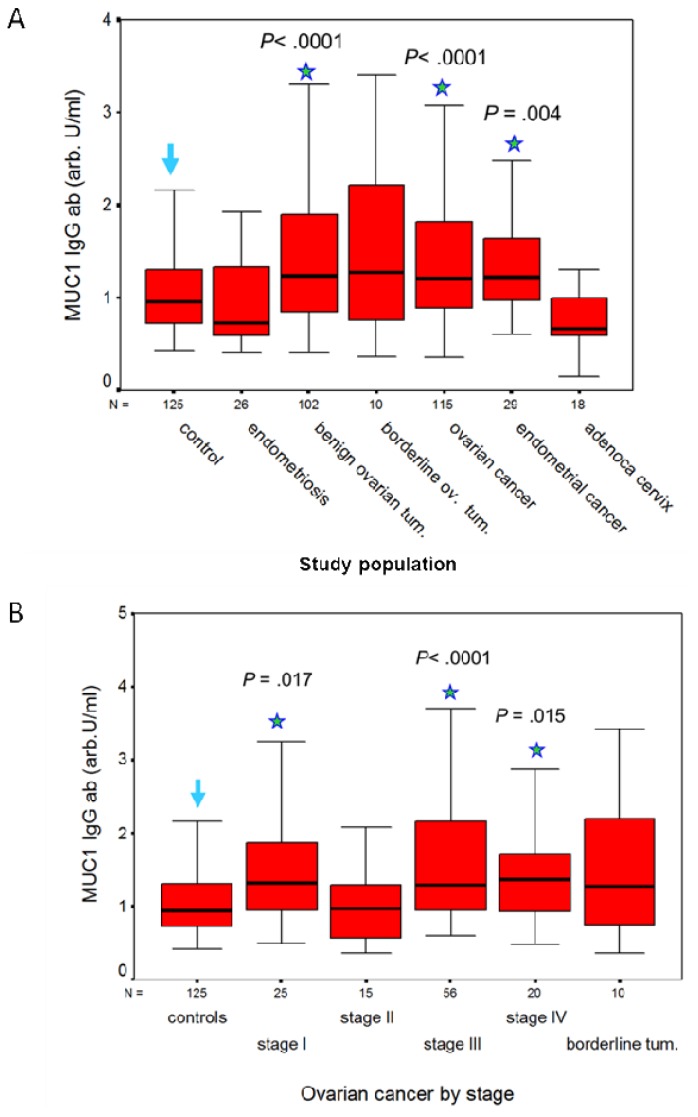
Box and whisker plots of MUC1 IgG ab levels in: (**A**) healthy controls, benign and malignant gynecological diseases; (**B**) ovarian cancer according to FIGO stage.

A humoral response to MUC1 is present in patients with adenocarcinomas of the genital tract, but not in patients with squamous cell carcinoma (SCC) of the cervix. We measured IgG and IgM antibodies to MUC1 in a total of 652 serum samples with the ELISA described earlier, and calculated their concentration against a 5-point huHMFG1 dilution curve [63]. Serum samples were obtained from 125 healthy women and before treatment from 527 patients with benign, premalignant and malignant gynecological diseases as detailed in Table 1. CA 15.3, MUC1 IgG and IgM ab results are contained in Table 1. MUC1 IgG ab levels were significantly higher in patients with benign ovarian tumors and in patients with ovarian or endometrial cancer than in healthy controls or patients with endometriosis (Figure 2A). By contrast, MUC1 IgM ab levels were significantly lower in patients with ovarian or endometrial cancer than in healthy controls (*p* < 0.001 and *p* = 0.001, respectively), in patients with endometriosis (*p* = 0.009 and *p* = 0.013, respectively), and in patients with benign ovarian tumors (*p* < 0.001 in both cases). No significant difference in MUC1 IgG and IgM abs was observed in ovarian cancer or in benign and borderline tumors in relation to histological type. MUC1 IgG ab levels in patients with ovarian cancer according to FIGO stage are illustrated in Figure 2B. MUC1 IgG levels were significantly lower in FIGO stage II (the least represented population) than in the other stages, but did not differ significantly between the other three stages (Figure 2B). CA15.3 levels were significantly higher (*p* < 0.001) in ovarian cancer compared to benign and borderline ovarian tumors, and increased with FIGO stage (Table 1). In patients with squamous cell cancer (SCC) of the cervix, MUC1 IgG and IgM ab levels did not differ significantly from levels measured in women with CIN lesions of the cervix. No significant difference in MUC1 ab levels was found between stages, or according to negative or positive lymph node status. SCC of the cervix expresses MUC1 [6], but little is known on the frequency of this expression and, to our knowledge, nothing on the degree of glycosylation of MUC1 in SCC of the cervix. Abs to MUC1 were also low in the patients with adenocarcinoma of the cervix, but this may be due to the smaller numbers studied (18 *vs.* 95). CA 15.3 levels, however, were significantly higher (*p* = 0.002) in the patients with SCC than in women with CIN lesions of the cervix (Table 1). As established for other carcinomas, CA 15.3 levels are related to tumor load in SCC of the cervix [66]. No correlation was found between MUC1 IgG or IgM ab levels and MUC1 (CA 15.3) assay results in any of the groups studied.

Circulating MUC1 IgG abs have also been reported in patients with head and neck squamous cell carcinoma, multiple myeloma, and colorectal adenoma and cancer [55,64,67,68,], and are been evaluated as part of a panel of autoantibodies to TAA for the early diagnosis of cancer [69-71]. Furthermore, initial studies suggest an association between GM allotypes, genetic markers of IgG heavy chains, and levels of MUC1 IgG abs that may have an implication for immunotherapy [72,73].

### 2.3. Antibodies to MUC1 and Disease Outcome

An association between elevated levels of MUC1 IgG abs and a benefit in survival was observed in patients with lung, pancreatic, breast and gastric cancer. MUC1 IgG ab levels measured in 30 patients with non-resectable non-small cell lung cancer were lower (*p* < 0.001) than those of healthy subjects (N 60). A significantly higher one-year survival rate (90.9% *vs.* 21.1 %, p < 0.001) was observed in patients with high MUC1 IgG than in patients with low ab levels [74]. Pretreatment MUC1 IgG but not MUC1 IgM ab levels measured in 36 patients with invasive ductal pancreatic carcinoma (2 Stage I, 10 Stage II, 8 Stage III, and 16 Stage IV) correlated significantly with survival time (*p* = 0.0004). Survival of patients with MUC1 IgG OD ≥ 0.3 was significantly longer than in those with MUC1 IgG OD < 0.3 (*p* = 0.008). MUC1 IgG remained significant (HR, 0.03; 95%CI, 0.003–0.289; *p* = 0.0024) after multivariate analysis for disease stage, age and gender [75]. We analyzed the incidence of naturally occurring abs to MUC1 in 154 early breast cancer patients (52 Stage I and 102 Stage II) and related their presence in pretreatment serum to outcome of disease. A positive test result was defined as MUC1 IgG or IgM ab levels equal to or higher than the corresponding rounded median results obtained in the total breast cancer population (0.7 OD and 0.8 OD for MUC1 IgG and IgM, respectively). A positive test result for both MUC1 IgG and IgM abs in pretreatment serum was associated with a significant benefit in disease-specific survival in stage I and II (*P* = 0.0116) breast cancer patients. Positive IgG and IgM MUC1 ab levels had additional prognostic value to stage (*P* = 0.0437) in multivariate analysis. Disease-free survival probability did not differ significantly. However, stage II patients who tested positive for MUC1 IgG and IgM abs and who relapsed had predominantly local recurrences or contralateral disease, as opposed to recurrences at distant sites in the patients with a negative humoral response (*P* = 0.026) [65]. A positive correlation (*p* = 0.0001) between circulating Thomsen-Friedenreich (TF) IgG and MUC1 IgG abs was found in patients with early stage gastric cancer. High levels of these abs were significantly associated with a benefit in survival of patients with gastric cancer. The association was stronger for TF IgG than for MUC1 IgG abs (*p* = 0.003 and *p* = 0.037, respectively) [76]. A natural immune response to MUC1 seems insufficient to affect the course of disease in patients with epithelial ovarian cancer. No association with disease outcome in relation to MUC1 IgG abs was observed in 199 patients with epithelial ovarian cancer randomized to the control arm in a phase III clinical trial evaluating intraperitoneal treatment with a radiolabelled murine anti-MUC1 MAb [77]. However, similarly to what we had previously observed in breast cancer [65], the percentage of patients with elevated MUC1 IgG ab levels was higher in early than in late stage disease, suggesting a certain measure of control on disease spread [77].

### 2.4. Antibodies to MUC1 and Immune Surveillance

Recent epidemiological studies describe an association between conditions that induce abs to MUC1 and risk for ovarian cancer, suggesting a protective effect of the immune response on the development of the disease [78,79]. In this respect we found that women at hereditary high risk of breast/ovarian cancer with a BRCA1/2 mutation had significantly lower levels of MUC1 IgG ab (*p* = 0.003) than age-matched healthy controls. In contrast to our previous findings in sporadic breast cancer, no elevated MUC1 IgG abs were seen in women at hereditary high risk who developed breast cancer. These results suggest that, by strengthening immune surveillance, prophylactic immunotherapy with MUC1 substrates may be a strategy to reduce the risk of breast/ovarian cancer in BRCA1/2 mutation carriers [80].

### 2.5. Antibodies to MUC1 Glycopeptides

We found early on that natural humoral responses to MUC1 peptides go hand in hand with humoral responses to MUC1 glycopeptides, and that sera from carcinoma patients react more strongly with triple TR TnMUC1 (GalNAc-MUC1) glycopeptides than with the corresponding naked peptides [81]. Recently, seromic profiling of colorectal cancer patients with a glycopeptide microarray identified abs to aberrant glycopeptides derived from MUC1 and MUC4 that may be of use for colorectal cancer screening [82,83]. Antibodies to aberrantly glycosylated MUC1 were measured using a microarray platform of 20mer MUC1 glycopeptides in 395 patients with early breast cancer. Presence and levels of abs to the MUC1 glycopeptides were significantly higher in breast cancer patients than in controls. High levels of serum abs to core3MUC1 (GlcNAcβ1-3GalNAc-MUC1) and to STnMUC1 (NeuAcα2,6GalNAc-MUC1) were associated with a benefit in metastasis free survival (*p* = 0.032) but not in overall survival, without independent prognostic value in multivariate analysis [84]. However, core 3 glycans are not found either on normal or on malignant breast tissue suggesting that immune reactivities to core 3 glycans are either not related to breast cancer or result from epitope mimicry. The study of Blixt and colleagues [84] is part of a European multicenter study based on three large serum banks. Results of the latter study presented recently at the 11th Workshop on Mucins in Health and Disease on sera taken from healthy women who subsequently developed breast cancer did not give any support for the assumption that abs to MUC1 glycopeptides may be used as diagnostic markers [85]. This is not surprising considering the heterogeneity of circulating MUC1 and the polyclonality of the response to it. Furthermore, the presence of natural abs to MUC1 in healthy individuals and their relatively low incidence in patients with cancer will lead to diagnostic markers with a low specificity and sensitivity. However, availability of synthetic glycans and glycopeptides and the use of microarrays will make it easier to dissect immune responses to heterogeneous glycoproteins such as MUC1. This will contribute to further define the specificity of abs best associated with a benefit in disease outcome and pinpoint towards the optimal MUC1 substrates for vaccination.

### 2.6. Assays Used in the Studies

Several factors pertaining to the setup of the ELISAs used in the studies here reviewed may have influenced the results obtained, and could explain conflicting results between them. In contrast to the peptide bound to BSA or HSA [63,86], MUC1 peptides directly bound to the plate fail to efficiently detect IgG abs [61,62]. The majority of studies use relatively low serum dilution (≤1:40) for screening for anti-MUC1 abs. This may lead to unreliable results particularly in the case of IgM responses, which are more subject to interference by natural antibodies of broad specificity than are IgG responses. Furthermore, screening for MUC1 IgM and IgG at a high serum dilution avoids interference of natural human anti-Gala(1,3)Gal antibodies, which directly cross react with deglycosylated MUC1 expressed on cancer cells and MUC1 tandem repeat peptides [87]. Furthermore, many studies rely only on a negative and a positive control to test for assay variability. Whereas this may be sufficient for small populations that can be tested in one day [75], a standardized assay is necessary for studies performed in large populations. A standard curve constructed with a reference sample introduced into the assay assures the reliability of results obtained and permits their quantification [63,74,88,89].

### 2.7. Epitope Mapping of Natural Abs to MUC1

Natural humoral responses to MUC1 are polyclonal, and the abs recognize several sequences on the MUC1 peptide repeat. Whereas the immunodominant region for the mouse is the PDTR sequence [90], the minimal epitope sequence most frequently recognized by human natural MUC1 IgG abs is RPAPGS, followed by PPAHGVT and PDTRP (see Figure 1 for the TR sequence) [81]. In agreement with these results, a previous study had shown that human abs generated by EBV-immortalized B-cells from tumor-draining lymph nodes of ovarian cancer patients recognized the APPAH sequence in 4 out of 5 cases, and only in one case an epitope that contained the PDTR sequence [91]. Furthermore, two Fab antibodies specific for MUC1 selected from a large naïve phage ab library recognized, respectively, the PAPGS and RPAPGSTAPPAH sequences [92]. In another study, IgG ab purified using a triple TR peptide from serum of a patient with a myosarcoma expressing MUC1 recognized a PPAHG epitope region, and the PPA sequence was also common to IgG abs purified against the patient's MUC1 or MUC1 from patients with advanced breast cancer [93]. The observation that natural MUC1 IgG abs, in contrast to abs induced by MUC1 peptide vaccination, bind more strongly to GalNAc modified MUC1 peptides than to naked peptides indicated that a MUC1 glycopeptide would be a better vaccine substrate than a naked peptide [81]. The findings of Graves and colleagues [93] that abs purified against tumor-associated MUC1 display a wider range of polyclonality than abs purified against a MUC1 peptide, and are more discriminatory between normal and tumor-associated MUC1 also support this notion. Furthermore, a decameric glycopeptide with the sequence SAPDT(GalNAc)RPAPG binds to the MHC classI allele HLA* 0201, suggesting that MUC1 tandem repeat glycopeptides are capable of activating both helper and cytotoxic T-cells [94]. The variant PESR motif that replaces the PDTR in up to 50% of the TR is often in concert with a Pro to Ala replacement in the TAPPA sequence (Figure 1) [24]. Whereas binding to MUC1 IgG abs from nonmalignant control subjects are preferentially directed to variant repeats and exhibit variable binding to variant and invariant MUC1 glycopeptides, MUC1 IgG abs in patients with adenocarcinoma recognize preferentially the invariant PDTR peptide, and bind equally well to all glycopeptides [95]. The variant PESR motif shows higher conformational flexibility than the PDTR motif, and the concerted TR sequence variation of TAPP to TAPA is associated with reduced glycosylation densities of the variant repeats. These characteristics could explain the observed differential responses in non-malignancy and malignancy. Studies on the processing of *O*-glycosylated MUC1 in the MHC class II pathway show that glycans are not removed during processing by DC and that the activation of helper T-hybridoma cells is dependent on site-specific effects of glycans attached to the major cathepsin L processing site, indicating that not only glycosylation itself but also the site of glycan substitutions are important in the design of MUC1 glycopeptide vaccines [96,97]. No difference was found in cathepsin L-catalyzed cellular processing of invariant and variant peptides [98]. Thus, the peptide-directed humoral response to variant and invariant TR clusters would be largely dependent on the sites and average density of *O*-glycosylation. The pattern of reactivity exhibited by serum samples from patients with adenocarcinoma can be explained by the reduced glycan chain lengths on tumor-associated MUC1, which facilitate the accessibility to the invariant peptide core, and enable immune responses to it [95].

## 3. Anti-Tumor Effect of Abs to MUC1

A plethora of studies on the association between tumor associated MUC1, tumor progression and the immune system suggest that abs to MUC1-N may be instrumental in (1) preventing metastasis; (2) counteracting immune suppression; and (3) effecting antibody-dependent cell-mediated cytotoxicity (ADCC).

### 3.1. MUC1 Abs and Prevention of Metastasis

MUC1 overexpression and the aberrant cell surface glycosylation associated to it play a role in cancer progression by altering adhesion and anti-adhesion of tumor cells, therefore favoring invasion of lymph and blood vessels by tumor cells, and extravasation of tumor cells at distant sites. Abs bound to the (glyco)peptide or glycan epitopes on MUC1-N may restore cell-cell interaction and prevent tumor progression by inducing capping or clustering of MUC1 and exposing cell adhesion molecules covered by MUC1, as observed *in vitro* with MAbs to the MUC1 TR [99]. MUC1-N towers above the plasma membrane, shielding the cancer cell and obscuring smaller cell surface molecules involved in cell-cell adhesive interactions. MUC1 overexpression strongly decreases cell-cell and cell-matrix interactions, and prevents epithelial cell aggregation by interfering with integrin-mediated adhesion and with E-cadherin- mediated cell adhesion [99-102]. Furthermore, the heavily glycosylated extracellular domain of MUC1 can simultaneously mediate and block binding to adhesion molecules with some molecular specificity, illustrating the dual role of MUC1 in tumor invasion [103]. MUC1 is a ligand for the intercellular adhesion molecule 1 (ICAM-1), a member of the immunoglobulin superfamily [104,105]. ICAM-1 plays an important role in leukocyte migration from the capillary to the site of tissue damage, an ability that could be used equally well by the tumor cell to reach the circulation [106]. In the same way that binding of MUC1 to ICAM-1 is inhibited by a MAb to the MUC1-N peptide core [104], natural or induced abs to it could prevent adhesion of cancer cells to ICAM-1 on the cell surface of fibroblasts and endothelial cells, impeding migration of cancer cells away from the tumor and into the circulation.

TACA, which are abundantly expressed on the MUC1 molecule, play an important role in adhesion/anti-adhesion processes that promote tumor progression [107,108]. Studies in colon carcinoma cell lines have shown that sialyl-Lewis ×/a residues mediate the adhesion of malignant colonic cells to E-selectin [109,110]. STn and Tn on MUC1 expressed on tumor cells contribute to perineural invasion in pancreatic cancer by binding to myelin-associated glycoprotein (MAG), a member of the Siglec family of sialic acid-binding lectins expressed on oligodendrocytes and Schwann cells [111]. Galectin-3, a member of soluble lectins that bind β-galactoside-containing glycans, is elevated in the circulation of patients with advanced metastatic cancer. Interaction of galectin-3 with cancer-associated MUC1 via TF expressed on circulating tumor cells induces a clustering of MUC1 on the cell surface that leads to the exposure of epithelial adhesion molecules, promoting cancer cell adhesion to endothelium and tumor cell aggregation leading to the formation of tumor emboli [112,113]. Blocking of these binding sites by abs to MUC1 glycopeptides could contribute to limiting disease progression.

### 3.2. MUC1 Abs may Hinder MUC1-Mediated Immune Suppression

Soluble MUC1 inhibits adhesion of MUC1-expressing cells to ICAM-1 suggesting an immunosupressive role for the MUC1 shed into the tumor microenvironment and the high levels of circulating mucin often found in advanced stage disease [104]. The ICAM-1 binding site on MUC1 lies within the peptide core, exposed in the cancer-associated, aberrantly glycosylated mucin, and MUC1 binds to domain 1 of ICAM-1 [104,105,114]. ICAM-1 plays a role in leukocyte transendothelial migration, and its counter-receptors are LFA-1 (CD11a/CD18) and Mac-1 (CD11b/CD18), two members of the integrin family. LFA-1 is required for a broad range of leukocyte functions, including T cell-mediated killing, T-helper and B-lymphocyte responses, and ADCC. Mac-1 is involved in cell-cell and cell-substrate adhesive interactions, and is a receptor for complement 3 [115,106]. Proliferation of T-cells submitted to polyclonal stimuli is inhibited by MUC1 added to the culture, and the inhibition can be reversed by exogenous Il-2 [116-118]. High amounts of MUC1 shed into the environment of tumors could block ICAM-1 binding sites on immune effector cells and contribute to the anergic state of tumor-infiltrating lymphocytes.

TACA are also implicated in immune suppression and tumor growth. Sialoadhesin, a macrophage restricted adhesion molecule frequently expressed in macrophages infiltrating breast tumors, binds to sialic acid on MUC1 expressed by the breast cancer cell line MCF-7 [119], an interaction that may be of importance not only for macrophage infiltration of tumors, reputed to be a source of cytokines and angiogenic factors which promote tumor growth, but also, paradoxically, to enable tumor cell killing by macrophages [120].

### 3.3. MUC1 Abs Mediate Antibody-Dependent Cell Cytotoxicity (ADCC)

Numerous studies suggest that ADCC is an important mechanism of action for several MAb in cancer therapy [121]. To the present, ADCC had not been directly demonstrated *in vivo*, but inferred from the requirement for IgG Fc receptors (FcγR) in tumor rejection in mice, and suggested by the association between genomic polymorphism of FcRγIIIb and clinical results obtained with MAb therapy [122]. A recent study has shown that tumor cells form stable conjugates *in vivo* with FcR-γ chain-expressing macrophages and neutrophils in mice treated with a chimeric anti-Tn MAb (Chi-Tn MAb), but not in control MAb-treated mice. The contact zone between tumor cells and ADCC effectors (host peritoneal cells) accumulate actin, FcγR and phospho-tyrosines, and these *in vivo* formed “ADCC-synapses” are organized in multifocal supra-molecular activation clusters (SMACs). These results show that *in vivo* ADCC mediated by macrophages and neutrophils during tumor rejection by Chi-Tn MAb involves a novel type of multifocal immune synapse between effectors of innate immunity and tumor cells [123].

A humanized IgG1 MAb to MUC1, huHMFG1, and serum samples from breast cancer patients with high levels of MUC1 abs induced by vaccination with MUC1 peptides mediate ADCC *in vitro*. NK cells were the principal effector cells for MUC1 ab-dependent killing, which could be inhibited by serum samples of carcinoma patients with high levels of MUC1 [124,125]. Furthermore, invariant NKT (iNKT) cells, which are known to activate NK cells, and TLR ligands, which activate both iNKT and NK cells, enhance ADCC mediated by huHMFG1 *in vitro*, suggesting that TLR agonist treatment may improve the efficacy of antibody-based therapies [126].

**Figure 3.**
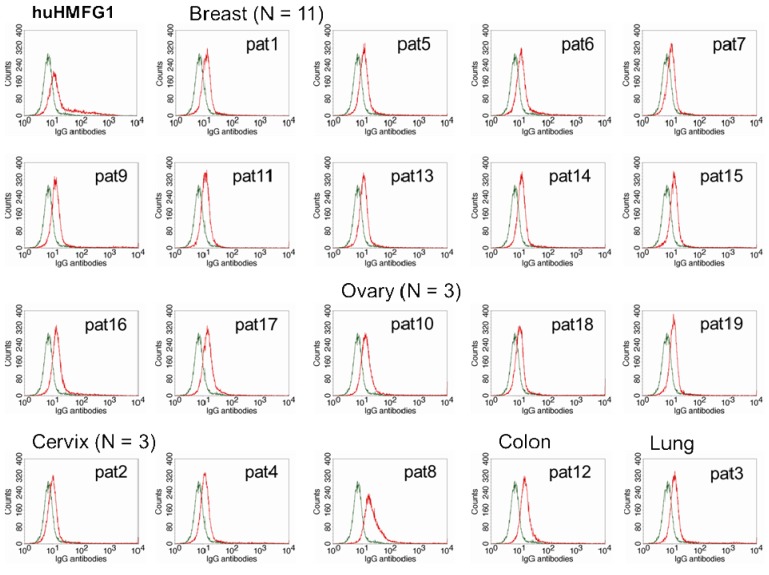
Flow cytometry analysis of binding of huHMFG1, a humanized IgG1 MAb to MUC1, and IgG purified from serum samples of 13 patients with adenocarcinoma of the breast, ovary, uterine cervix, colon and lung with high anti-MUC1 IgG levels to ZR75-1, a breast cancer cell line expressing MUC1 (red line), and control IgG1 (green line).

**Figure 4.**
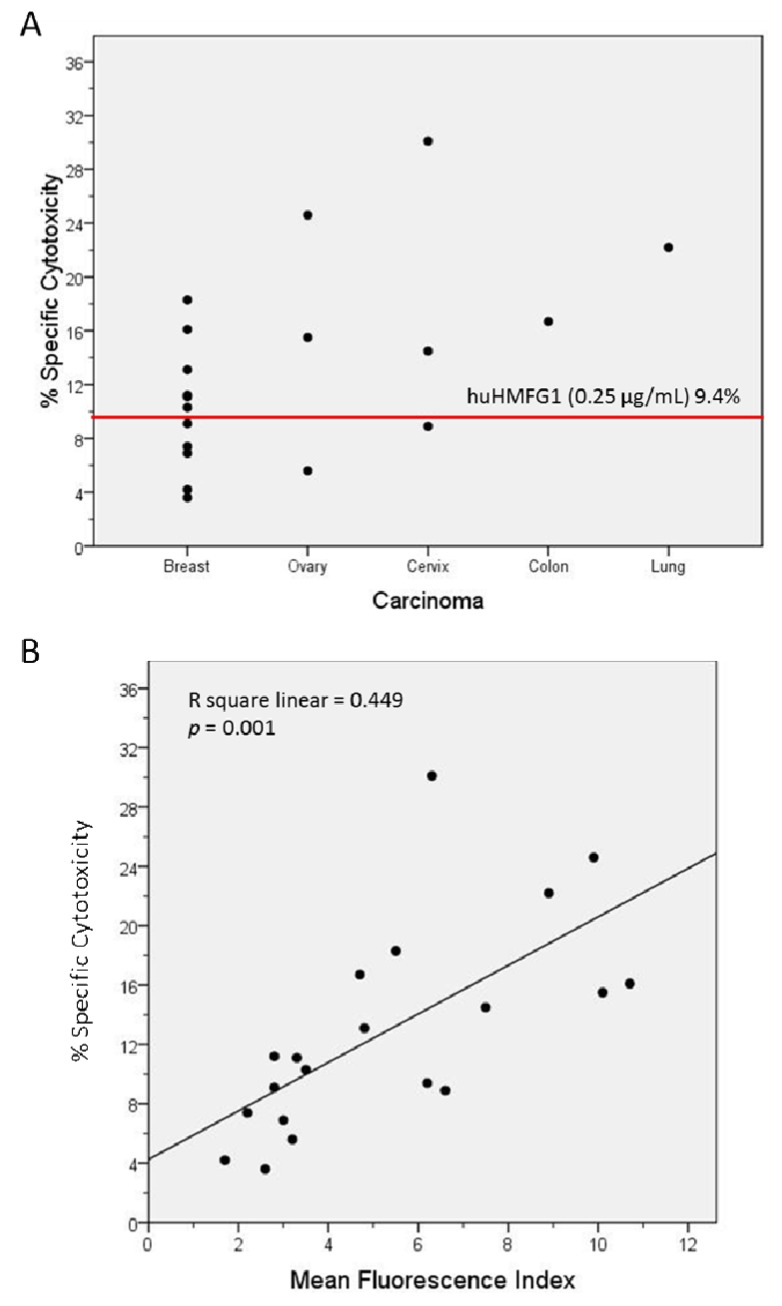
(**A**) % specific cytotoxicity mediated by IgG purified from serum samples of 13 patients with adenocarcinoma of the breast, ovary, uterine cervix, colon and lung with high anti-MUC1 IgG levels tested with an ADCC assay; the red line marks the level of cytotoxicity mediated by huHMFG1; (**B**) linear regression analysis of the % specific cytotoxicity and the mean fluorescence index obtained with huHMFG1 and the patients' samples.

The optimal IgG subclasses to mediate ADCC are IgG1 and IgG3. IgG subclasses analyzed in MUC1 in 55 healthy controls and 26 breast cancer patients with high levels of abs to MUC1 were mostly IgG2, but also IgG1 and IgG3 [81]. Natural MUC1 abs bind to MUC1-expressing cancer cell lines [74,75]. We found that the ability of natural antibodies to bind to tumor cells overexpressing MUC1 correlated significantly to their capacity to mediate ADCC of the same tumor cells. We purified IgG from serum from 19 patients with adenocarcinoma (11 breast, three ovarian, three cervical, one colon, and one lung cancer) with high levels of IgG ab to MUC1 and tested their ability to bind to MUC1 expressed on a breast cancer cell line and to mediate ADCC. Total IgGs were purified from sera using HiTrap Protein G HP columns. MUC1 IgG ab levels of the purified samples were determined by ELISA, and their concentration calculated against a 5-point huHMFG1 dilution curve [63]. Unfortunately, IgG subclasses were not analyzed in this group of patients. Binding property of 10 ng of purified IgG from patient sera or huHMFG1 was assessed by FACS analysis using ZR-75-1, a breast cancer cell line that overexpresses MUC1. Seven out of 19 purified IgG samples showed higher binding capacity to the tumor cell line than huHMFG1 (Table 2 and Figure 3). Their ability to mediate antibody-dependent tumor cell killing of ZR-75-1 was determined by a ^51^Cr release assay, as previously described [125]. Twelve out of 19 samples showed a higher capacity to mediate ADCC than huHMFG1 (Figure 4A), suggesting that natural antibodies may be more effective in mediating tumor cell killing than huHMFG1 despite their lower binding capacity in some cases. Intensity of binding to ZR-75-1 correlated significantly (R Sq. linear = 0.449, p = 0.001) with tumor cell killing (Figure 4B). ADCC may play an important role in immune surveillance by destroying easily accessible isolated disseminated tumor cells present in the circulation and the peritoneal cavity that could eventually lead to recurrence of disease.

## 4. MUC1-Targeted Immunotherapies that Induce Abs to MUC1

MUC1 has been extensively studied as a target antigen for the immunotherapy of cancer, and some modest clinical responses have been reported [127]. A large number of vaccine constructs based on MUC1 tested in clinical trials are geared to induce cellular responses, and they either do not induce abs to MUC1, the humoral responses are very weak or are not monitored during the trial [88,128,129]. MUC1 vaccines that induce abs are largely based on MUC1 TR peptides or glycopeptides coupled to a carrier protein and administered with an immunological adjuvant [131].

### 4.1. MAb Therapy

With the exception of a recently approved dendritic cell vaccine for the treatment of prostate cancer, all currently approved immunotherapies directed to distinct tumor-associated antigens are based on the passive administration of MAbs [121,132]. Anti-idiotypic abs to MUC1 have been observed in the context of passive administration of MUC1 murine MAbs [133]. In ovarian cancer patients, a benefit in survival was observed after intraperitoneal administration of radiolabelled HMFG1, a murine monoclonal antibody to MUC1 [134]. The therapeutic effect may have been due to the radiolabel, but studies showed induction of an immune network cascade in the patients receiving the drug, with development of anti-idiotypic antibodies and cellular immunity [135,136]. To study the therapeutic effect of radioimmunotherapy targeting MUC1, a large multicenter phase III clinical trial was carried out in a total of 447 patients with epithelial ovarian cancer who had attained a complete clinical remission after cytoreductive surgery and platinum-based chemotherapy with yttrium-90-labeled murine HMFG1 (^90^Y-muHMFG1). The patients were randomized to receive a single intraperitoneal administration of ^90^Y-muHMFG1 plus standard treatment or standard treatment alone. The study did not show a disease-free (DFS) or overall survival (OS) benefit in patients treated with ^90^Y-muHMFG1 [136]. However, patients that received the drug developed IgG abs to MUC1 that had an impact on DFS (*p* = 0.036) and OS (*p* = 0.043) in univariate, but not in multivariate analysis (*p* = 0.070 and *p* = 0.135 for DFS and OS, respectively). In spite of the treatment being suboptimal to induce an effective immune response, Kaplan-Meier analysis showed a significant benefit in disease outcome in patients with highest levels of anti-MUC1 IgG in the patients that received a single administration of ^90^Y-muHMFG1 but not in the patients that received standard treatment [77].

### 4.2. Vaccination with MUC1 Substrates

The first phase I clinical trial with MUC1 peptides was carried out in 13 patients with metastatic breast cancer. Patients were vaccinated intradermally or intramuscularly with a 20 aa MUC1 peptide conjugated to diphtheria toxoid in increasing doses (four groups) at 2 week intervals (three injections). No toxicity was found and only weak antibody and T cell proliferative responses to MUC1 were seen [138]. A 16 aa MUC1 peptide conjugated to KLH plus DETOX adjuvant administered to 16 patients with metastatic breast cancer induced ab responses in only three patients, and CTL activity against MUC1-expressing tumor cell lines in seven patients [139]. A vaccine composed of a 100 aa MUC1 peptide administered with SB-AS2 adjuvant (a mixture of MPL and QS-21 in an oil-in-water emulsion) to 16 patients with resected or locally advanced pancreatic cancer induced weak MUC1-specific humoral and T-cell responses in some patients [140].

Several clinical trials have been carried out with a MUC1 fusion protein coupled to mannan or to oxidized mannan in patients with adenocarcinoma [127]. Twenty-five patients with advanced metastatic carcinoma of breast, stomach, colon or rectum were vaccinated subcutaneously in increasing doses with a MUC1 fusion protein (5 TR) coupled to mannan. The vaccine induced strong MUC1 IgG responses, and to a lesser extent cellular responses. The response was polyclonal, and most of the antibodies reacted with the STAPPAHG and PAPGSTAP sequences of the tandem repeat [86]. Another study evaluated the influence of cyclophosphamide and the route of injection on mannan-MUC1 peptide immunization in 41 patients with metastatic or locally advanced adenocarcinomas, mainly of the breast, colon and rectum. Overall 60% of the patients had high MUC1 IgG titers, with the intraperitoneal route yielding 10-fold higher responses. Cyclophosphamide was of no benefit. Disease progressed in most patients, and remained stable in five patients [141]. A pilot phase III was carried out in 31 Stage II breast cancer patients randomized to receive oxidized mannan-MUC1 or placebo. Of the patients receiving the vaccine, nine out of 13 had measurable abs to MUC1 and four out of 10 had MUC1-specific T cell responses. After a follow up of 8.5 years since the start of the trial the recurrence rate in the placebo group was 27%; none of the patients in the immunotherapy group had a recurrence (*p* = 0.029), suggesting that in early breast cancer MUC1 immunotherapy is beneficial [142].

The Memorial Sloan-Kettering Cancer Center has carried out numerous phase I/II clinical trials over the years testing the immunogenicity and safety of MUC1 peptides and glycopeptides conjugated to KLH administered with a saponin, QS21, first as monomeric vaccines and later as part of polyvalent vaccines with several TACA antigens [131]. Two MUC1 peptides, a 30 aa and a 33 aa covalently conjugated to KLH and administered with QS-21 as immunological adjuvant, were tested in two phase I clinical trial in, respectively, nine and 10 breast cancer patients with no evidence of disease (NED) after treatment of a recurrence, elevated CEA or CA 15.3 levels or initially unresectable stage III disease post therapy. The vaccine was administered in five doses of 100 μg of MUC1 peptide each on week 1, 2, 3, 7 and 19. The vaccine had low toxicity and induced high MUC1 IgG and IgM antibody responses that were polyclonal in nature [81,143,144]. Serum samples from the vaccinated patients mediated ADCC [124]. In contrast to natural anti-MUC1 IgG antibodies from breast cancer patients that react more strongly with glycosylated than with naked MUC1 peptides, the induced antibodies tested in ELISA against defined GalNAc-MUC1 glycopeptides showed progressively diminishing reactivity with increasing glycosylation of the peptide [81]. These results indicated that a MUC1 glycopeptide would be a better vaccine than a naked peptide. A series of phase I trials with MUC1 glycopeptides conjugated to KLH induced IgG and IgM abs that bind better to MUC1-expressing cells than the abs induced by vaccination with MUC1 peptides [145]. A heptavalent vaccine containing GM2, Globo-H, Lewis Y, Tn(c), STn(c), TF(c), and Tn-MUC1 individually conjugated to KLH and mixed with QS21 was tested in 11 patients with epithelial ovarian, fallopian tube, or peritoneal cancer. The vaccine was well tolerated and induced abs to five of the seven antigens. Humoral responses were largely IgM against each antigen with the exception of Tn-MUC1 where both IgM and IgG responses were induced [146]. Complement-dependent cytotoxicity (CDC) against a breast cancer cell line was seen in seven of nine patients, but possibly not due to MUC1 abs, as these do not mediate CDC [147]. A similar hexavalent vaccine that was tested in 30 high-risk patients with prostate cancer in a phase II setting induced abs against two or more antigens in all patients, but ab titers were lower than those seen in previous trials with each monovalent vaccine. One possible explanation for this would be the increased levels of the carrier protein KLH in the polyvalent vaccine compared to monomeric vaccines [148,149]. Progress in carbohydrate synthesis has led to new vaccine constructs that may be more effective in inducing an immune response: a unimolecular multivalent vaccine containing various carbohydrate antigens incorporated into a single peptide backbone and conjugated to KLH following an improved KLH conjugation protocol that enables a significant increase in epitope ratio of the KLH conjugate induced strong ab responses in mice [150]. Following the same principle a hybrid vaccine construct containing a unimolecular pentavalent glycopeptide domain (Globo-H, GM2, sTn, TF, and Tn) covalently linked to a MUC1 TR peptide has been synthesized [151]. A possible next step would be to link the glycopeptide construct to a MUC1 glycopeptide, instead of a naked peptide.

Several promising fully synthetic MUC1-based constructs stand in line for phase I clinical studies that will test their safety and immunogenicity. Selective immune responses have been induced in preclinical studies with fully synthetic MUC1 glycopeptide vaccines that include tetanus toxoid as carrier protein, or incorporate a TLR2 ligand lipopeptide, Pam3CysSerK4, in the construct [152-156], and new constructs conjugated by click chemistry to give multivalent MUC1 glycopeptide vaccines are still to be tested for immunogenicity [157]. These self-adjuvanting, multicomponent vaccines avoid anti-carrier immune responses while incorporating the necessary structural features for evoking an essential class switch from low-affinity and short lived IgM abs to high-affinity IgG abs [156]. Before being able to proceed to clinical trials in human subjects, further preclinical testing of these new compounds is necessary to demonstrate tumor rejection and protection in animal models, and, in particular, the safety and toxicity of the TLR2 ligand.

## 5. Conclusions

Innate and adaptive immunity work in concert, and an optimal cancer vaccine should exploit the abilities of both arms of the immune system [157]. Although cytotoxic T cells are indispensable to achieve anti-tumor responses in advanced disease, abs to tumor-associated antigens are ideally suited to address minimal residual disease and may be sufficient to exert adequate immune surveillance in an adjuvant setting, destroying tumor cells as they arise or maintaining occult disease in an equilibrium state [158,159]. Hematogenous dissemination occurs early on in the disease in most types of carcinomas, and the presence of tumor cells in the bone marrow at the time of primary surgery is associated with an unfavorable disease outcome [160]. Minimal residual disease persists despite adjuvant therapy in a majority of patients with primary breast cancer up to four years following surgery [161]. A natural humoral immune response to MUC1 not only may have a favorable influence on disease outcome in patients with carcinoma, but may also protect against the development of the disease. Antibodies to MUC1 may exert a beneficial effect by curbing metastatic spread and immune suppression mediated by MUC1, and can be induced by MUC1-based vaccines. The idea of prophylactic vaccines that exert immune surveillance on cancer in subjects at high risk of malignancy is very attractive. Whereas vaccine constructs for cancer immunotherapy will need to include T-cell epitopes or Toll-like receptor agonists to effectively recruit the immune system, a prophylactic effect may well be achieved already with simpler vaccine constructs combined with a strong adjuvant. A key requirement for the use of prophylactic vaccines based on modified self-antigens, as would be the case with MUC1 glycopeptides, is that they should not induce autoimmunity to healthy tissue [162,163]. Another prerequisite would be that the same or similar vaccines exert an anti-tumor effect in patients with cancer. These vaccines have to be tested as early on in the disease as possible [164,165], and necessarily in a large study population, which means prolonged and costly clinical trials. In summary, immunotherapy that relies on humoral responses to achieve a clinical effect is most effective in patients with a low tumor burden (minimal residual disease), and is of limited use in advanced metastatic disease. The stronger immune response and the immunologic memory that are associated with active immunotherapy, administered in an adjuvant setting, would provide a constant surveillance mechanism to protect cancer patients from recurrence of disease. Vaccination with MUC1 glycopeptides may be even more effective if initiated before primary surgery. An anti-tumor immune response already present at surgery may contribute to limit a possible dissemination of tumor cells caused by surgical manipulation. Furthermore, vaccination can be continued immediately after surgery, and provide protection against disease dissemination during the healing process until it is possible to start chemotherapy.

## Figures and Tables

**Table 1.** Anti-MUC1 antibodies and MUC1 (CA 15.3) levels in benign and malignant gynecological diseases.

**Study population**	**N 652**	**MUC1 IgG (Arb.U/mL) Median (range)**	**MUC1 IgM (Arb.U/mL) Median (range)**	**CA 15.3 (U/mL) Median (range)**	CA 15.3 P*
Healthy controls	125	0.95 (0.43–5.70)	2.7 (0.96–15.78)	17 (1–48)	
Controls with CIN lesions of the cervix	134	1.04 (0.51–5.58)	3.09 (1.08 –12.03)	17 (4–54)	
Endometriosis	26	0.73 (0.41–7.26)	2.94 (1 –8.85)		
Benign ovarian tumors	102	1.23 (0.40–20.49)	2.73 (0.79–15.22)	15 (5–50)	n.s.
Borderline ovarian tumors	10	1.28 (0.37–3.41)	2.16 (0.70–9.22)	16 (5–24)	n.s.
Ovarian carcinoma	113	1.19 (0.36–7.11)	1.92 (0.51–9.91)	44 (5–1282)	<0.001
- Stage I	25	1.12 (0.50–4)	2.23 (0.68–9.21)	25 (5–350)	
- Stage II	14	0.84 (0.36–2.09)	2.35 (1.02–8.30)	28 (9–166)	
- Stage III	56	1.29 (0.61–7.11)	1.78 (0.51–9.91)	58 (9–1282)	
- Stage IV	20	1.37 (0.48–2.98)	1.89 (0.63–4.66)	71 (8–1067)	
Endometrial carcinoma	29	1.22 (0.61–2.48)	1.81 (0.67–9.33)	21 (5–65)	n.s.
Adenocarcinoma of the cervix	18	0.67 (0.15–2.85)	2.48 (1.07–15)		
Squamous cell carcinoma of the cervix	95	0.92 (0.42–13.58)	2.92 (1.03–10.26)	18 (4–117)	0.002¶

* Mann-Whitney U test, CA 15.3 levels in each group compared to levels in healthy controls.

¶ Mann-Whitney U test, CA 15.3 levels compared to levels in controls with CIN lesions.

**Table 2.** Intensity of binding to ZR-75-1 (FACS) and tumor cell killing (ADCC) of IgG purified from serum samples of patients with carcinoma.

**Patient**	**Tumor type**	Mean fluorescence index *	**% specific cytotoxicity** ¶
1	Breast	1.7	4.2
2	Cervix	6.6	8.9
3	Lung	8.9	22.2
4	Cervix	6.3	30.1
5	Breast	3.5	10.3
6	Breast	2.8	9.1
7	Breast	2.8	11.2
8	Cervix	7.5	14.5
9	Breast	10.7	16.1
10	Ovary	10.1	15.5
11	Breast	3.0	6.9
12	Colon	4.7	16.7
13	Breast	2.2	7.4
14	Breast	4.8	13.1
15	Breast	5.5	18.3
16	Breast	3.3	11.1
17	Breast	2.6	3.6
18	Ovarian	9.9	24.6
19	Ovarian	3.2	5.6
huHMFG1		6.2	9.4

* Mean fluorescence index (MFI) is calculated as the mean fluorescence intensity of the purified IgG/mean fluorescence intensity of the control IgG, measured by flow cytometry.

¶ % specific cytotoxicity is calculated as the difference between % ^51^Cr release in wells with purified IgG and control antibody.
